# Scholar in the *SEPR* spotlight: Ian Douglas

**DOI:** 10.1007/s42532-020-00051-6

**Published:** 2020-06-05

**Authors:** Ian Douglas

**Affiliations:** School of Environment, Education and Development, University of Manchester, Manchester, M13 9PL UK

**Keywords:** Ian Douglas, Applied geography, Active geography, Tropical geomorphology, Urban environment, International collaboration, Graduate students, Socio-ecological practice and research

## Abstract

In this reflective essay of intellectual autobiography, I respond to a series of questions the journal editor Wei-Ning Xiang asked about my 55-year journey from applied geography to socio-ecological practice research. These are (1) what and/or who had inspired your career most in geography and socio-ecological practice research? (2) Throughout your 55-year academic journey, did you ever reorient your ambitions in scholarly pursuit, or even reinvent yourself in your academic life? What motivated you in each of these instances? (3) How do you measure success in your work? Among many accomplishments, what are the top three that you are most proud of? (4) From your personal experience, what would be the most important attributes for a well-lived, fully realised, and meaningful life? Do you have any tips for maintaining work-life balance? (5) Do you have any specific advice for younger scholars in geography and socio-ecological practice research? (6) What are the three most interesting images reflecting turning points in your career? I hope that my experiences and insights showcased in this essay are helpful to the younger generations of geographers and socio-ecological practice researchers.

## What and/or who had inspired your career most in geography and socio-ecological practice research?

### My grandfather, Professors Jean Tricart and Gilbert White

**Fig. 1:**
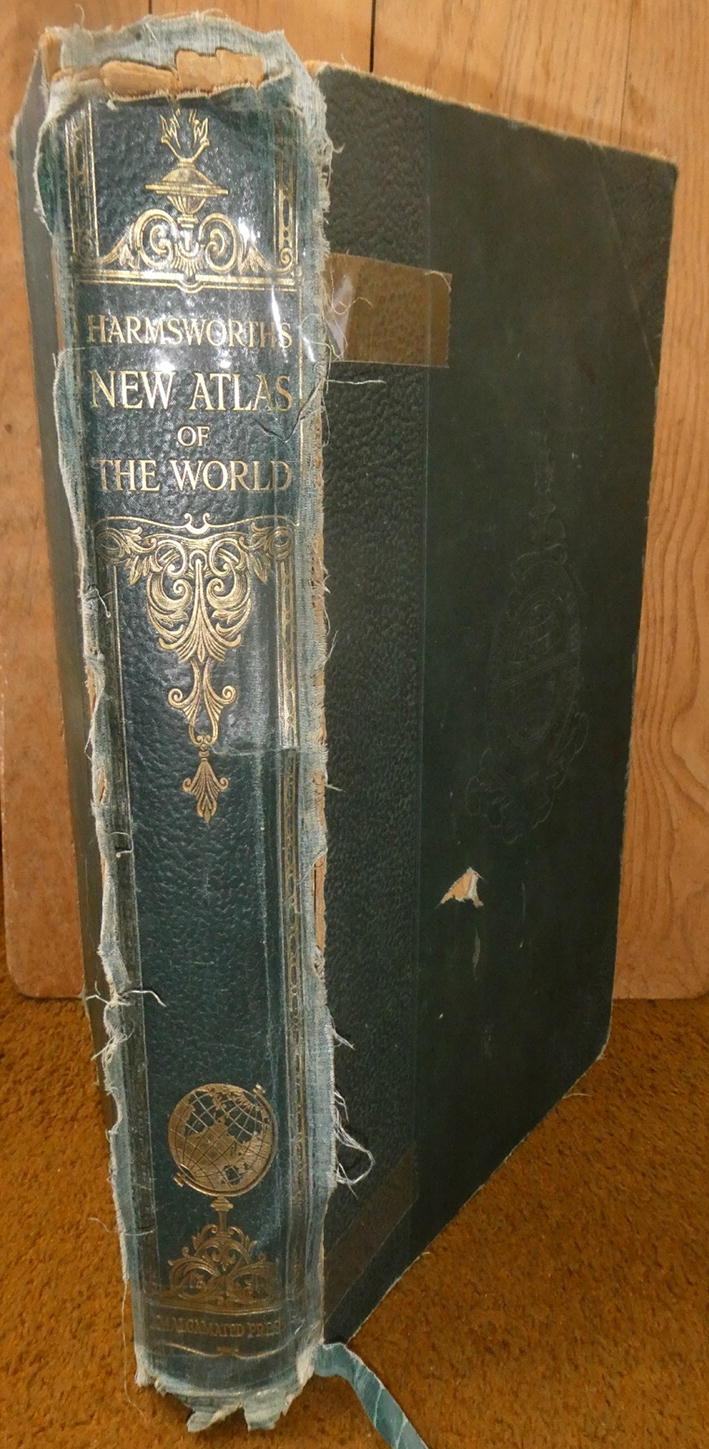
Ian’s much used 100-year old Harmsworth’s World Atlas (photo by Ian Douglas)

The earliest influence on my development as a geographer was my grandfather’s copy of Harmsworth’s New Atlas of the World (Hammerton 1920), which he showed me whenever our family visited him at his country cottage during the early 1940s. The pictures and maps in the Atlas revealing the diversity of the world and the contrasts between countries fascinated me, awaking a desire to learn more about distant places. When I was 8 years old, Grandad gave the Atlas to me and I proudly carried it home. It remains on the bookcase next to my desk (Fig. 1). By 1958 when, after 2 years’ compulsory national service in the Royal Artillery, I went to Balliol College, Oxford University to study Geography, I started to think about what I should be believing and doing. As a physicist, my Father believed strongly in a scientific approach to the world. National service reinforced my view of the world as an unequal place with much deprivation and unkindness. I began to believe that privileged people like me should be working to make the world a better place.^1^

**Fig. 2:**
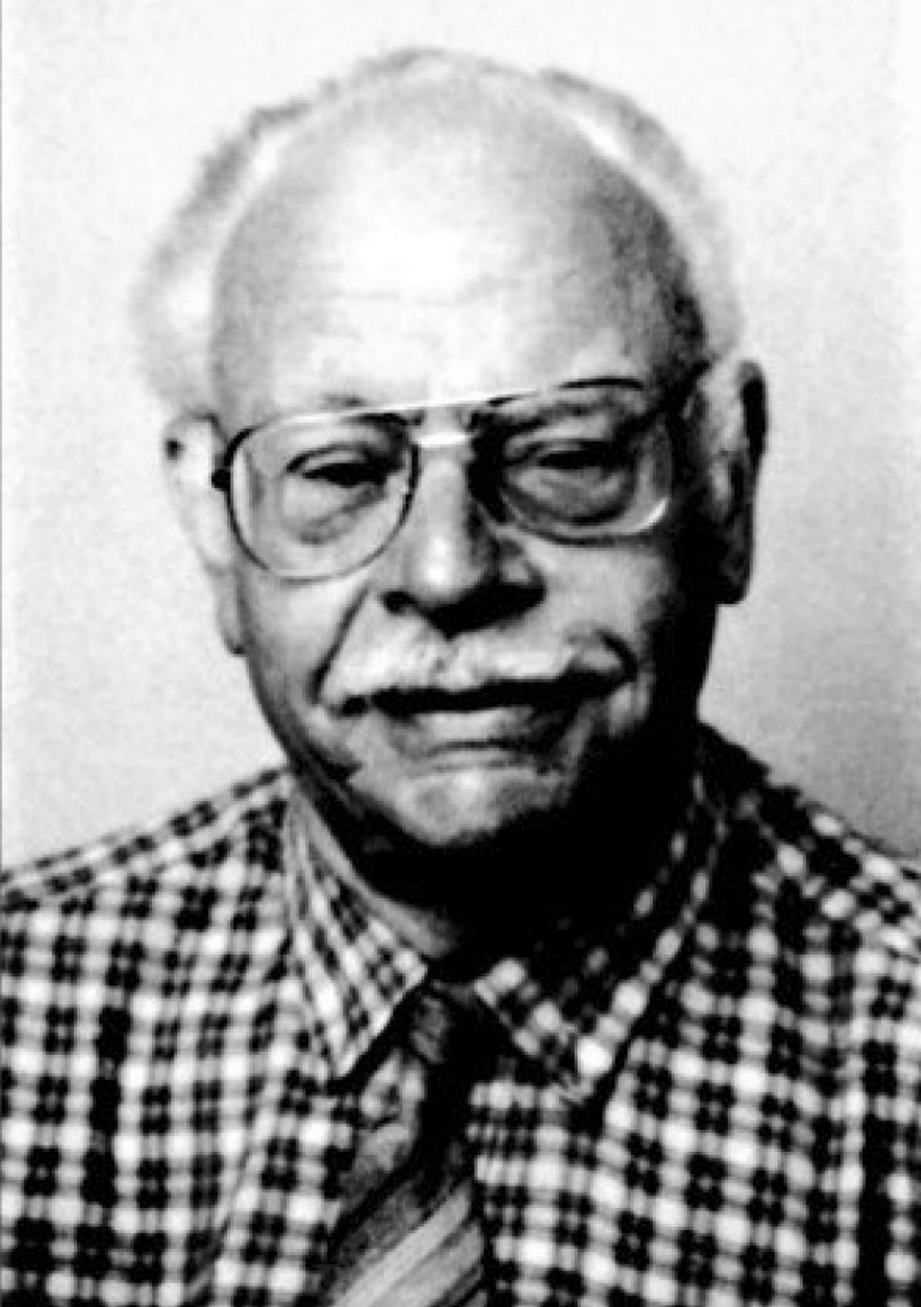
Professor Jean Tricart 1920–2003 (from Mainguet 2003 p.193)

The Oxford course taught us to write, criticise, and argue rigorously. The tutorial system was a wonderful training in marshalling evidence and arguing a case. However, it was all somewhat literary and remote from current issues. The type of practical work done by leading geographers during and after the Second World War (Stamp 1960) seemed more urgent than defining geographical regions. So also did the applied geomorphology practised by French geomorphologist Jean Tricart (Fig. 2), so well expressed in a *Revue de Géomorphologie dynamique (Review of Dynamic Geomorphology)* editorial:

Idealism is a luxury that we are less and less able to afford. Time is pressing. Soils are eroding, while people are hungry. Immense tasks await the coming generations of geomorphologists. [Cailleux and Tricart 1961 (in French), p.2; English translation by the author]^2^

I took the first step in my ambition to practice applied geography in Liège, Belgium seeking advice from Omer Tulippe whose geography was described as: “*Above all active, full of life, applicable and applied*” [Schmitz 2003 (in French), p. 99; English translation by the author].^3^ He emphasised the contrast between geography as a pure science and the new active, applied geography contributing to land use planning (Tulippe 1956, p. 173). His colleagues Frans Dussart and José Sporck were highly supportive. José later edited a splendid atlas and text *Liège prepares its future* (Sporck 1980) that is a good example of the applied geography that inspired me during my time in the city.

Six months after being in Liège, I was in Strasbourg, France, attending the lectures of Professor Tricart; joining fieldwork on the glacial deposits of the foreland of the French Jura; and subsequently an undergraduate field course in the southern French Alps. It was an eye-opener. I had not seen such landscapes before. I had not been told to stand before a great exposure of folded strata and explain why it looked the way it did. I had not spent Saturdays walking along a trench being dug for an oil pipeline to see the stratigraphy exposed by the trench and attempting to make sense of what it revealed about Quaternary changes in the Rhine Rift Valley. Here were many insights that Oxford had not given me. I began to understand the importance of geomorphology in planning decisions and landscape management.

**Fig. 3:**
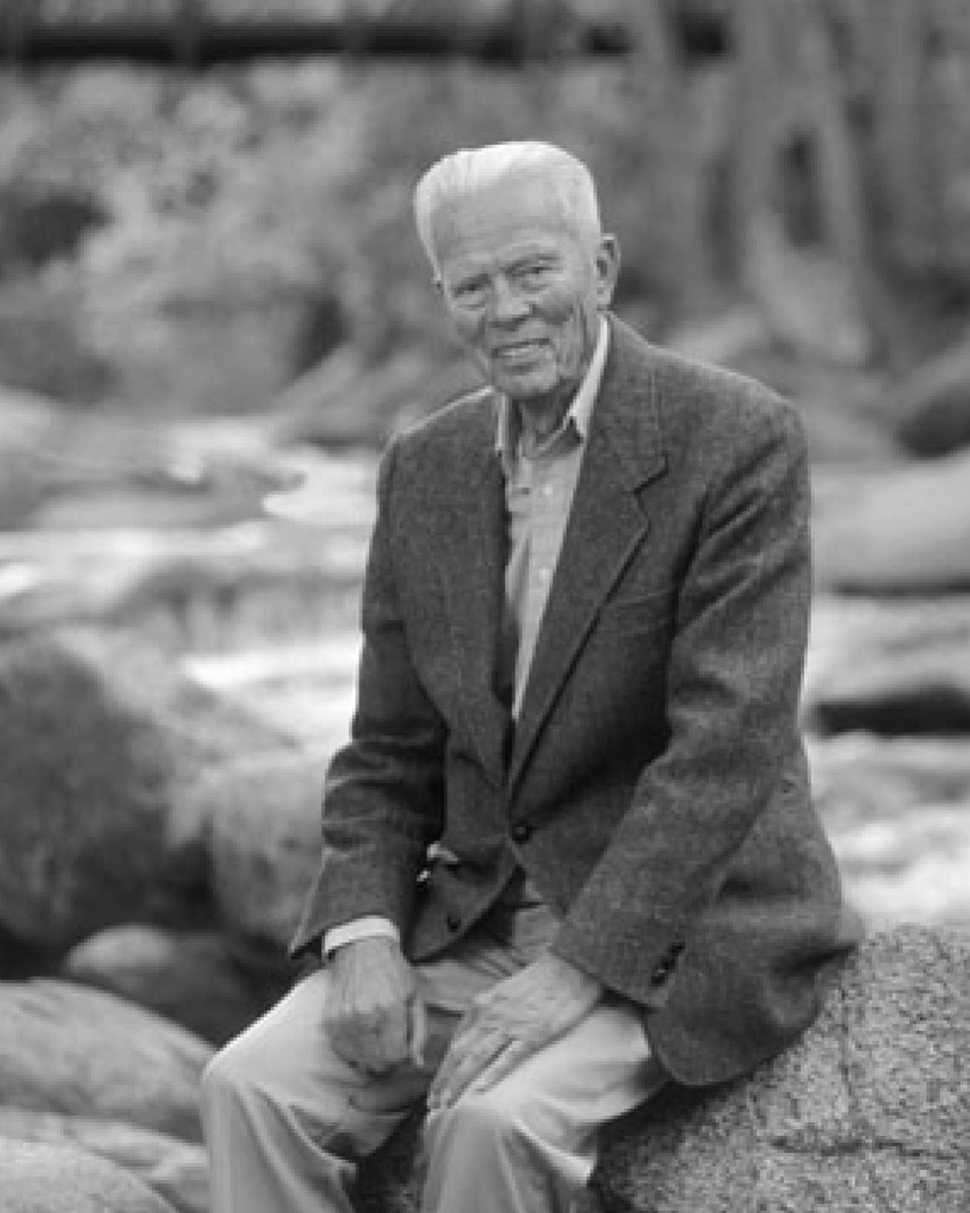
Professor Gilbert White 1911–2006 (photo by Ken Abbott UCB)

When I returned to Oxford, I met Gilbert White (Fig. 3), Professor of Geography at Chicago, whose pioneering work on floods and floodplain management had had a major influence on flood hazard strategies is the USA of America (White 1937, 1945; White et al. 1958). His lectures were inspirational; explaining how to study people’s perceptions and attitudes to floods and apply such understanding to flood plain management: an applied geography with a powerful mission of helping reduce human economic losses from flooding. I encountered Gilbert White later in my career and I believe he influenced my becoming involved with SCOPE [the Scientific Committee on Problems of the Environment of the then International Council of Scientific Unions (ICSU)]. Gilbert had been President of SCOPE, of which I later became Treasurer and Chair of its UK SCOPE Working Party.

### My experiences in Australia in the 1960s and 1970s

Moving to Australia in 1963 for Ph.D. work at the Australian National University (ANU) was a life changing step. The view of the Australian landscape with all its dead trees from the train from Sydney to Canberra revealed a different world from Europe: an ancient piece of Gondwanaland that would have to be interpreted in a different way from Western Europe. Fortunately the brilliant supervision and advice of Joe Jennings, Donald Walker, Harold Brookfield and Oskar Spate, all “Poms” who had permanently migrated from the UK to Australia, helped me to set-up research to test differences in denudation rates between the tropics and the temperate zones by measuring the sediment and solute loads of similar rocks in far North Queensland (latitude c 17°S) and southeast New South Wales (latitude c 35°S) (Douglas 1971). The work was made possible by generous assistance from many people in organisations such as the Snowy Mountain Hydroelectric Authority, CSIRO and the Queensland Irrigation and Water Supply Commission. Their readiness to assist was inspirational: I had to deliver a good thesis to all those who had helped me.

**Fig. 4:**
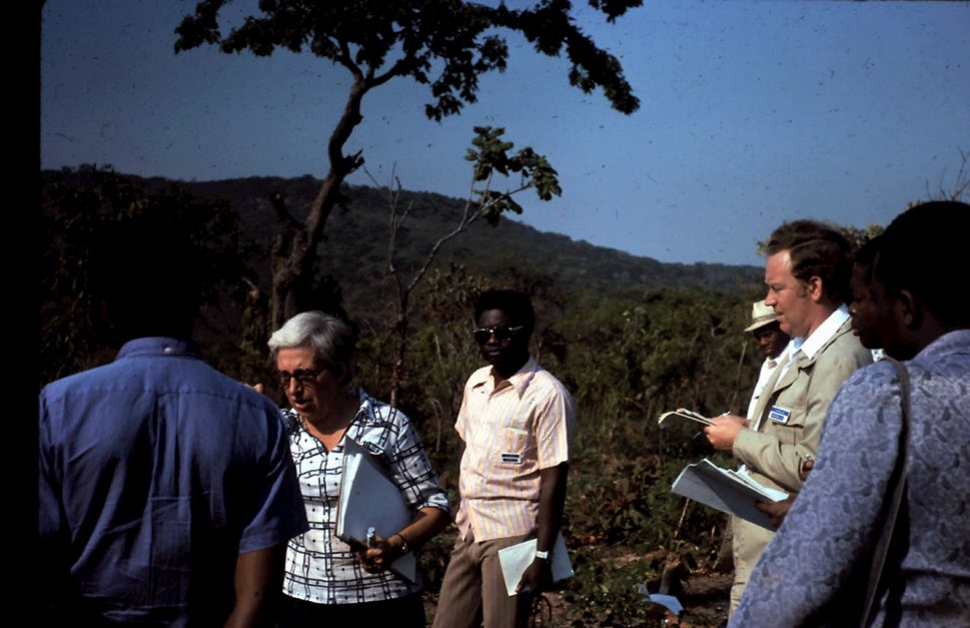
Ian (second from right) in a field discussion with S. Alexandre-Pyre (second from left) and local students in the field during a symposium on environmental geomorphology in the tropics in Zaire (now the Democratic Republic of the Congo) in 1975 (Photo by Michael Thomas)

In 1971 I became a Professor of Geography at the University of New England (UNE) in Armidale, New South Wales, Australia: a life changing big moment. I moved from a city (Hull) of some 250,000 population to one of 20,000 (Armidale), at the time, over 400 km from the nearest other university. Set-up at the instigation of local people to serve the rural communities and to research on rural industries, in addition to full-time students, the University taught external students by correspondence and occasional weekend schools. The Geography Department, a pioneer in distance learning for university geography, had a great record of applied geography serving local councils, examining problems ranging from flooding to rural development (Douglas et al. 1975). I replaced Gilbert Butland, the Foundation Professor of Geography, on many local and regional committees, so making direct contact with local business people, councillors and state politicians. I was frequently asked to undertake or organise projects to look at local issues across the North Coast and New England regions of New South Wales. Many students undertook applied projects that met local needs. I managed these requests for help, negotiating funding and reporting on outcomes. My colleagues were enthusiastic about these opportunities for applied geography. I also continued my activity in tropical geomorphology (Fig. 4).

### Michel Philipponneau, Zena Daysh, Rusong Wang, and Asenath Omwega

After 1975, I began to develop ideas for my book, *The Urban Environment,* and was inspired by Michel Philipponneau’s book on Rennes, France (Philipponneau 1976) in which he explains that his work was driven both by his geography and his membership of the Parti Socialist. He spent much time on citizen and political party committees as well as encouraging his geography students to investigate practical problems and solutions for the future planning of the city of Rennes. He helped to put his applied research into practice. For me, he went beyond applied geography to become an active geographer, advocating future directions for the communities in which he lived and worked. He put geography into practice. Today we might call him a socio-ecological practitioner.

**Fig. 5:**
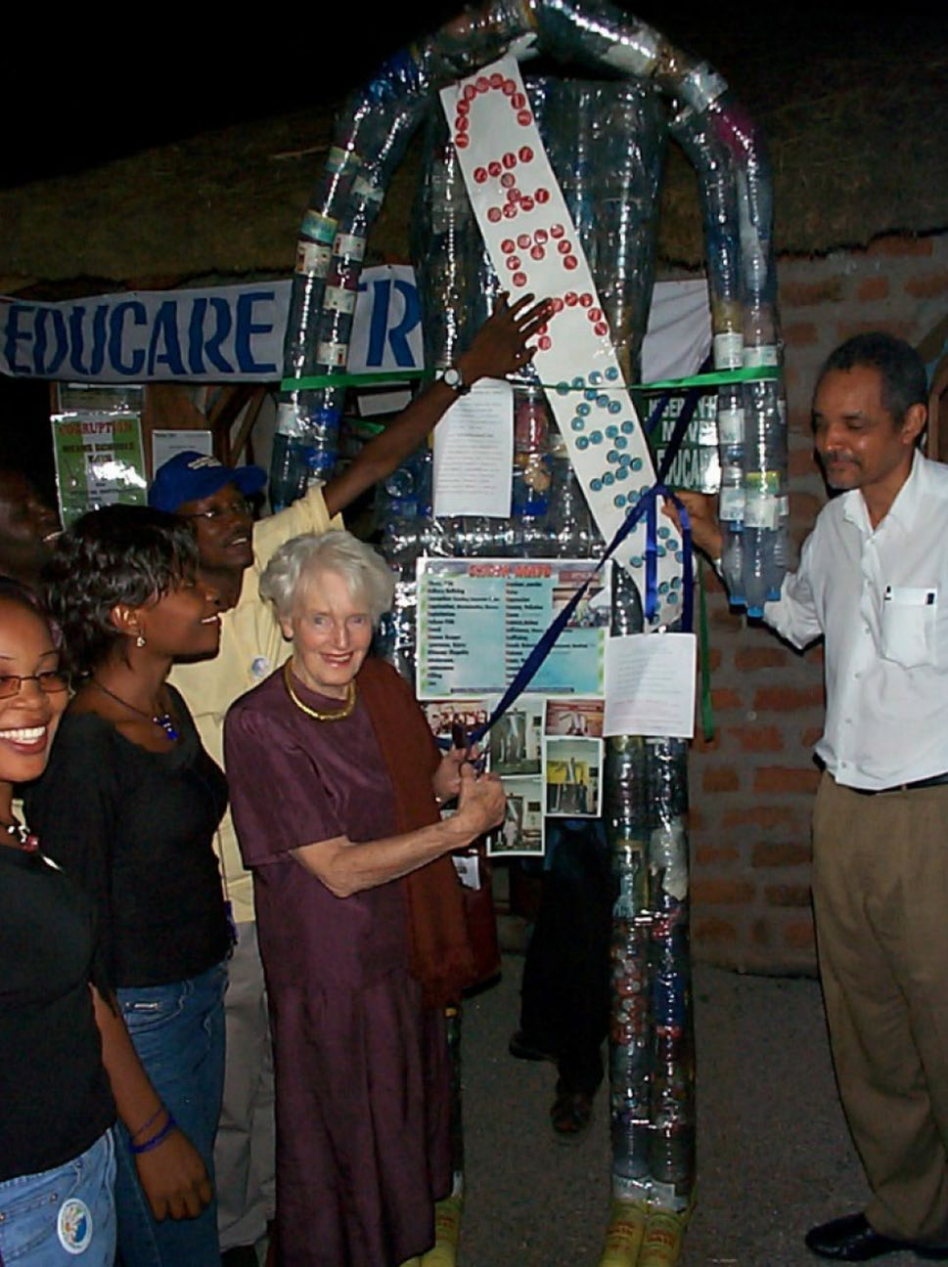
Zena Daysh CNZM (Companion of the New Zealand Order of Merit) at the People’s Forum of the Commonwealth Heads of Government Meeting in Abuja, Nigeria, 2003 with local students and NGO members (Photo by Ian Douglas)

In 1979, I returned to the U.K., and became a professor of geography at the University of Manchester (“Appendix 1”). Fourteen years later, Zena Daysh (Fig. 5), the dynamic founder of the Commonwealth Human Ecology Council (CHEC), contacted me. She persuaded me to be her local contact in Manchester to help her establish a one-day symposium before the UK Government’s international meeting on Local Agenda 21. Here I again interacted with local government, but also met Commonwealth High Commissioners and leading UN figures. I subsequently helped CHEC organise civil society sessions and UNHabitat work at Commonwealth Heads of Government meetings from 1995 to 2007. Zena Daysh introduced me to Prime Ministers and High Commissioners whom she had known for years. I vividly remember briefly talking to the Prime Minister of Kiribati in 1999 about the impact that climate change was already having on his islands. Zena Daysh influenced my thinking through her emphasis on “individual responsibility”, each of us taking charge of our own lives and trying to make the world a better place. CHEC has run many community development projects. I organised funding for rag-pickers in Ahmedabad, India; training courses on gender mainstreaming in Uganda; and alternative work for girls labouring in Indian limestone quarries. These activities involve much political jostling and persuasion.

**Fig. 6:**
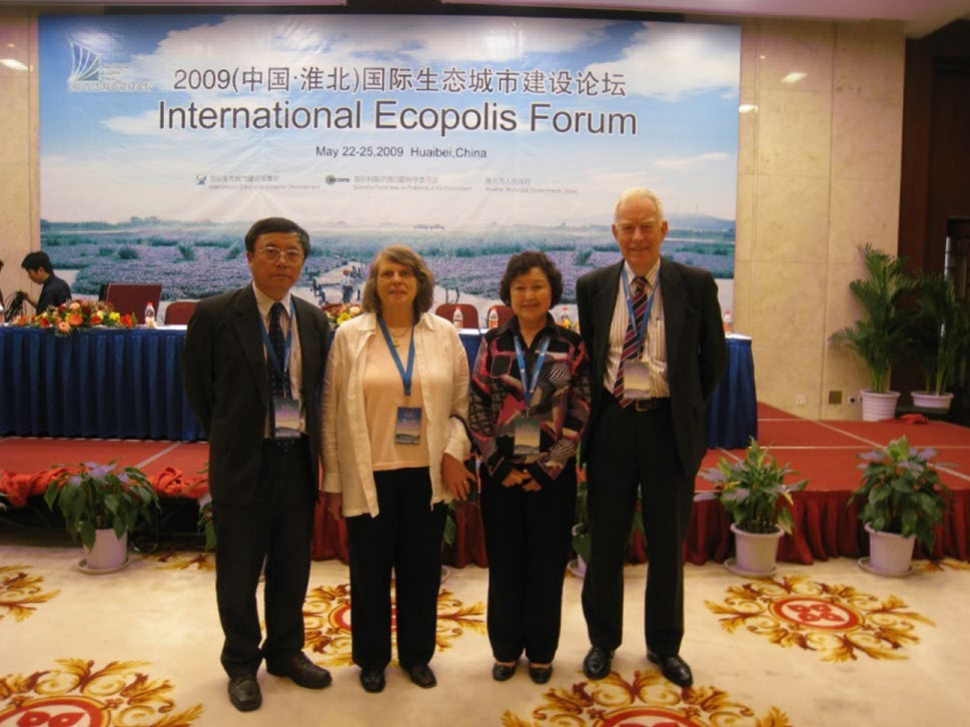
Left to right: Rusong Wang, Maureen Douglas (Ian’s wife), Yuanli Xue (Rusong’s wife), and Ian at the 2008 INTECOPOLIS meeting in Huaibei, China (official photo by the INTECOPOLIS Beijing Team)

**Fig. 7:**
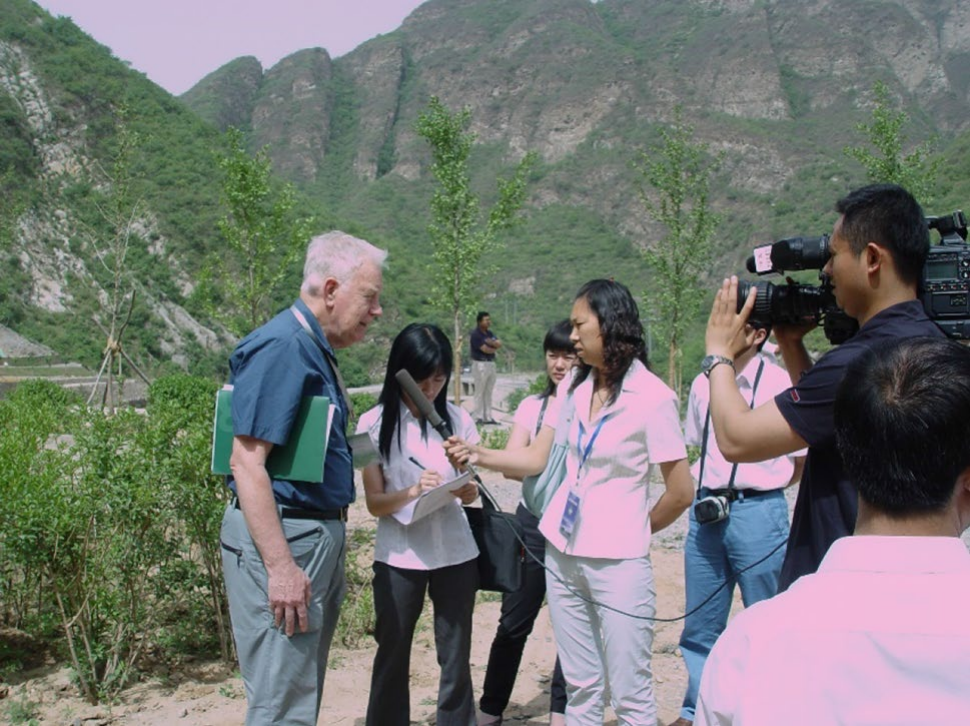
Ian being interviewed by a local television station at Mentougou, near Beijing, China (official photo by the INTECOPOLIS Beijing team)

**Fig. 8:**
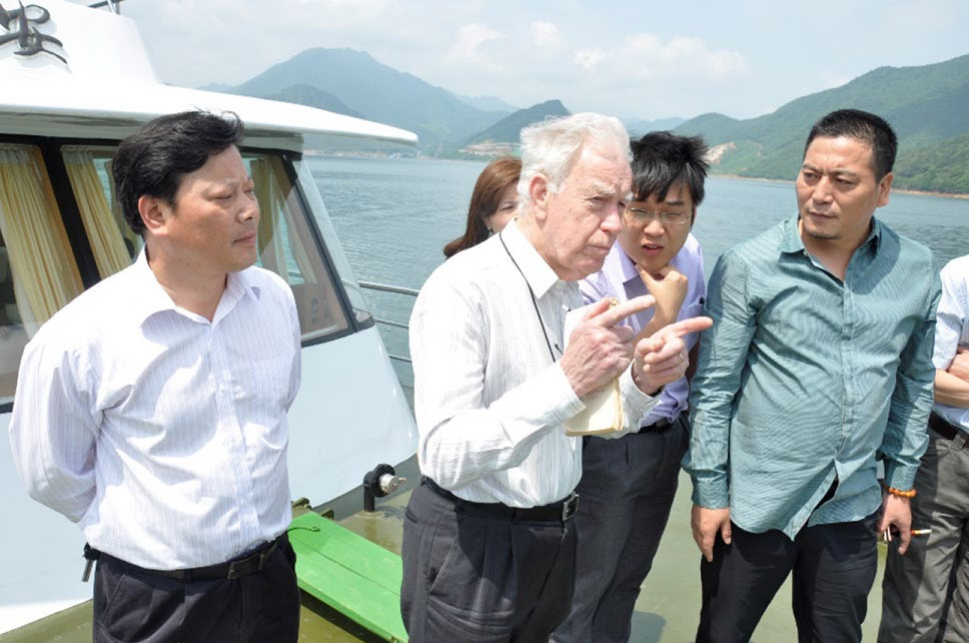
Ian discussing a possible future INTECOPOLIS project with local government offices and developer on the Quiandao Lake (thousand Island Lake) near Jian’de, Zhejiang Province, China (official photo by Jian’de local government)

The late Rusong Wang (Fig. 6) from the Chinese Academy of Sciences in Beijing, a key colleague in SCOPE, had a passion for human ecology and was totally engaged in socio-ecological practice. We worked on ecological urbanisation with Chinese local governments and researchers, such as the former coal-mining communities at Huaibei in Anhui Province and at Mentougou, west of Beijing, China (Fig. 7). Their land reclamation and redevelopment problems were similar to those in the areas west of Manchester, UK. We potentially made a difference by examining the reuse of coal-mining subsidence-caused lakes around Huaibei, with a Wigan wildlife trust expert going to Huaibei and the Mayor of Huaibei visiting Manchester and Wigan, to see local wetland restoration. Rusong Wang set-up INTECOPOLIS (“Appendix 2”) and I became its President (Fig. 8).

My graduate students have given me many opportunities to work in their countries. One of them, Asenath Omwega, then director of Action Aid’s African office in Nairobi, Kenya, was preparing a document for the Nairobi Climate Change Conference—November 2006 and COP 12. Action Aid had long argued that vulnerability to climate change was a major issue for Africa’s urban poor. Action Aid’s local teams assessed vulnerability in six African capital cities and met in Nairobi in 2006 where Asenath asked me to co-ordinate their information into a report: *Climate change, urban flooding and the rights of the urban poor in Africa* (https://www.actionaid.org.uk/sites/default/files/doc_lib/urban_flooding_africa_report.pdf) which was noticed by the world’s press. A paper developed from it (Douglas et al. 2008) is my most cited academic output. I am glad that a piece of active geography and socio-ecological practice has been so useful to so many.

## Throughout your 55-year academic journey, did you ever reorient your ambitions in scholarly pursuit, or even reinvent yourself in your academic life? What motivated you in each of these instances?

### A double transformation in the late 1960s and early 1980s

In 1966, I took a position in Geography at the University of Hull in the U.K. after I finished my Ph.D. at ANU. I spent my first 6 months in the job at the University of Malaya in Kuala Lumpur, Malaysia investigating erosion on granite to compare with that in north Queensland (Douglas 1967a, b). The key finding however was that erosion rates were far higher on urban housing construction sites than in the forest (Douglas 1968). Having recognised the importance of urban development in Kuala Lumpur for urban erosion, sedimentation and flooding, I became convinced that we ought to ensure that young urban people should understand the dynamics of the built environment. I went back to Kuala Lumpur in 1969–70 to make a more detailed analysis of urban erosion. This was really a double transformation: from a potential karst geomorphologist to an applied urban geomorphologist and a Southeast Asian environmentalist.

In Manchester, in 1979, I reconnected with the British Geomorphological Research Group (BGRG), becoming its Chairman in 1980. A joint UK-German workshop in Wurzburg led to a symposium on tropical geomorphology in Manchester and exchange visits to Heidelberg and Wurzburg, Germany. I organised the First International Conference on Geomorphology (Gardner 1987) which emphasised applied geomorphology, Jean Tricart being a keynote speaker. The interest in urban geomorphology of the 33 geomorphologists who came from China led to my giving a course on urban geomorphology at the SW Teachers’ University near Chongqing, China in 1988. I worked with the late Professor Gu Hengyue from Chongqing University on the sediment problems of China’s Yangtze and Yellow Rivers (Douglas 1989a; Gu and Douglas 1989; Douglas 1992; Douglas et al. 1993; Gu et al. 1993). This began the links with China, later enhanced by Rusong Wang, that greatly widened my perspectives on urban geomorphology and urban ecology.

### A second transformation in the 1990s

In 1992 I joined the UK Man and Biosphere (MAB) Urban Forum (now the UK Urban Ecology Forum). The membership helped me reinvent myself as an urban ecologist. Among other tasks, the Forum asked me to produce *The Routledge Handbook of Urban Ecology* (Douglas et al. 2010) whose Second Edition is due to appear in 2020 (Douglas et al. 2020). By 2009 this Professor of Physical Geography had become a leading figure in both Human Ecology and Urban Ecology. One could argue that they are really parts of geography. The editors of geography encyclopaedias have asked me to explain what these field cover (Douglas 2010, 2017). Human ecology has several connotations, but in the sense that I have been involved it is both a deep concern with individual responsibility and the care of individuals in their relationships not only with nature but with each other. Urban ecology on the other hand is more focussed on nature in the urban environment and people’s access to and enjoyment of that nature, its ecosystem services and its health and well-being benefits.

## How do you measure success in your work? Among many accomplishments, what are the top three that you are most proud of?

### The Australia-International Medal of the Institute of Australian Geographers

The 2006 citation for this medal recognised my major and ongoing influence on physical, environmental and applied geography in the Australian region and internationally, commencing with my pioneering ANU PhD thesis on rates of denudation in Eastern Australia (Douglas 1967b). This research explicitly recognised the magnitude of human impacts on erosion rates and soon extended to consider the impacts of urban environments on river systems as demonstrated by my texts on Humid Landforms (Douglas 1977) and The Urban Environment (Douglas 1983). Three sets of work, on wilderness, planning and tourism, illustrate how my colleagues and I influenced state and national government policies in the nineteen-seventies.

Becoming Head of the Department of Geography at the University of New England in Australia in 1972, I was anxious to put New England Geography in the national spotlight as a centre for excellence in environmental research. My 1973 presidential address to the Geography section of ANZAAS (the Australia and New Zealand Association for the Advancement of Science) on the future of Australia’s tropical rainforests gained national attention, including a radio programme, and led to a major Commonwealth Government funded project on Wilderness in Australia. I put together a team of a remote sensing specialist, a social geographer interested in tourism, and a biogeographer, with an experienced bushwalker graduate assistant. They mapped all wilderness areas of over 25,000 ha, at least 10 km in width, with a surrounding buffer zone of at least 25,000 ha. This was a big moment because the report (Helman et al. 1976) had an impact on state land use planning; with some of the areas they mapped becoming National Parks. (Sadly, many of those areas not far from Sydney were badly burned in the dreadful bushfires of December 2019–January 2020).

A critique of a much-changed New South Wales planning policy (Clark et al. 1975) written by our team led to the State Minister for Planning and Environment visiting the Department of Geography. Eventually the Department changed its name to Geography and Planning, teaching a professional planning course. I worked on the New England Advisory Council, the Technical Advisory Committee of the State Pollution Control Commission, and the NSW Land Conservation study group.

I also sent a comment on tourism to the House of Representatives Select Committee on Tourism and appeared as a witness before the Committee, which asked me to produce a report on the economic significance of tourism. The two colleagues who compiled this report (Pigram and Cooper 1977) later developed major academic contributions to the tourism literature (e.g. Cooper and Pigram 1984, Erfurt-Cooper and Cooper 2009, Wahab and Pigram 1997). This is an example is how an applied challenge can lead to new directions in serious academic work: there is not just a one-way route from theoretical to applied work; new ideas can come out of tackling a political or social issue.

I am proud of facilitating and supporting work of this kind, that lead to policy changes, increases in protected wilderness areas, improved planning procedures, and better tourism management.

### My book *The Urban Environment*

My 1983 book received little attention for its first 10 years, save for some outreach to conferences and teachers of geography (Douglas 1987, 1988, 1989b, c). Reviewers in the 1980s were sometimes critical. Human geographers saw the book as not being concerned with the processes studied by human geographers but explaining how human-driven environmental changes impact society (Johnston 1986). It was first listed in planning literature around 1990. It started to be cited in ecological studies after 2000. By 2005, environmental historians were being urged to read it in order to “give the subjects of site configuration, land creation and physical transformation, landscape, and soil attributes the attention they deserve” (Tarr 2005). The book has been used across disciplines and is seen as a pioneer contribution to urban physical geography and urban biogeography (Roberts 1985).

### My Malaysian legacy

I was fortunate enough to have many Malaysian graduates join me for Ph.D. work. The first of them, Low Kwai Sim, began a Ph.D. with me at Hull in 1967 (Low and Goh 1972). Eventually becoming a full Professor, she established her own environmental consulting company, while still teaching: a fine example of someone moving from applied geography to practice. She and my second Malaysian student, Zakaria Awang Soh, invited our family back to Malaysia in 1983, leading to further urban erosion work. Many of the students Kwai Sim and Zakaria sent to me are now professors at Malaysian universities.

In 1984, I joined the Royal Society’s South-East Asian Rainforest Research Programme (RSSEARRP), based mainly at the Danum Valley Field Centre in Sabah, Malaysian Borneo. The Programme’s key partner was Yayasan Sabah (the Sabah Foundation), a state semi-government body that used income from selective logging concessions for social welfare and education. The late Clive Marsh, their Conservation Officer, persuaded them to build a field centre catering for both environmental education and high-class research for the benefit of the people of Sabah. Clive was inspirational and enthusiastic. He helped me establish all the appropriate contacts. He let me know what the Sabahans wanted and I endeavoured to raise the funds to deliver the appropriate research.

In my first research grant application to the UK Natural Environment Research Council for funding for work at Danum Valley I sought funds to employ local Sabahan graduates as research assistants. My former Manchester colleague, Tom Spencer helped our post-doctoral researcher, Tony Greer, develop the field installations and mentor the young Sabahan graduates over their Master’s degree work. Later, as the Royal Society resident scientist at the Field Centre, Tony helped UK-based scientists develop good relations with all the Yayasan Sabah staff. He stayed in southeast Asia, working as an environmental consultant on aid projects, but sadly died in 2017. He was an academic who became a practitioner and changed the attitudes of plantation developers and forest exploiters.

**Fig. 9:**
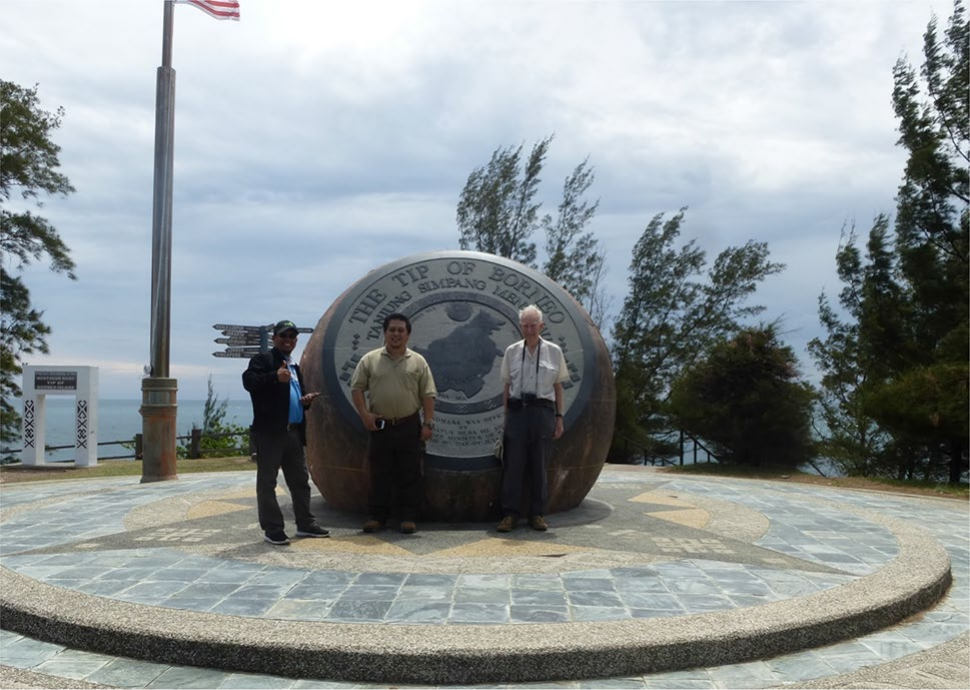
Right to left: Ian Douglas with Mustapa Talip (Universiti Malaysia Sabah) and Waidi Sinun (Yayasan Sabah), both former research assistants and graduate students at Manchester, at the “Tip of Borneo”, the northernmost point of Sabah, Malaysia in 2019 (photo by Maureen Douglas)

The Sabahan graduate students in our Danum research team in 1986–96 are now key local personnel (Fig. 9), continuing hydrological work at Universiti Malaysia Sabah (e.g. Bidin and Chappell 2006; Nainar et al. 2018), managing Yayasan Sabah’s conservation division, establishing new field stations, supporting the Sabah Parks and Wildlife Service, all being practitioners supporting social and environmental work. I encouraged them to present papers at international conferences (Sinun et al. 1992; Bidin et al. 1993). I recall one senior member of Yayasan Sabah saying how wonderful it was to see a Sabahan speaking at the Royal Society in London! I too am proud of their achievements.

Danum has supported Long-Term Ecological Research, with Yaysan Sabah support and many externally funded research projects, all of which have helped to develop local skills. For my wife and I, Malaysia is almost a second home, as is Australia. Later, I became involved more widely with the southeast Asia through research projects (e.g. Nguyen et al. 2008) and as a consultant to the Mekong River Commission on erosion in Cambodia, Laos, Thailand and Vietnam (Douglas 1997).

## From your personal experience, what would be the most important attributes for a well-lived, fully realised, and meaningful life? Do you have any tips for maintaining work-life balance?

I had many interesting conversations with the manager of a Belgian tour bus company serving the UK travel agency I worked for in the summers of 1958 and 59. He told me something that has stuck in my mind ever since: “You have to give something to get something”. The truth of this became apparent later: put something into an endeavour and usually something worthwhile comes out.

As my Mother said: “Do as you would be done by”.

Other attributes for a well-lived, fully realised and meaningful life:

1. Family comes first;
2. Work with others and learn from them;
3. Develop life-long friendships;
4. Welcome visitors from abroad;
5. Create opportunities for others;
6. Learn something new every day.

## Do you have any specific advice for younger scholars in geography and socio-ecological practice research (things to or not to do, books to read, etc.)?

1. Applied research can lead to new basic scientific understanding;
2. Fundamental hypothesis testing can lead to unexpected practical results;
3. Graduate students can be part of teams working on major programmes and should be encouraged to engage with local agencies and government departments;
4. Caring for graduate students (in the way that I was cared for at Oxford and the ANU) brings enormous, long-lasting rewards;
5. Finding ways around bureaucracies to achieve acceptable support for research colleagues and students pays dividends;
6. Engaging with the people living and working in the research area is essential;
7. Really getting to know the area you are studying; there is absolutely no substitute for ground truthing (what we used to call “getting your feet wet”).
8. Endeavouring to respond to requests from local governments, civil society organisations and environmental groups is worthwhile, but may be frustrating;
9. Seeing the unity of the subject, problems do not exist in silos;
10. Recognising where your skills can be useful and seeking help from others where you lack skills and information;
11. When the going gets tough, remembering the mission (To make the world a better place for our grandchildren’s children);
12. Not being upset when your grant application or paper submission is turned down: it happens to us all. (I always say to myself that they will reject the submission. If they say yes, I can be really happy! If no, then I just send off the next submission!)

## What are the three most interesting images reflecting turning points in your career?

### The Guil Valley in the French Alps, France

**Fig. 10:**
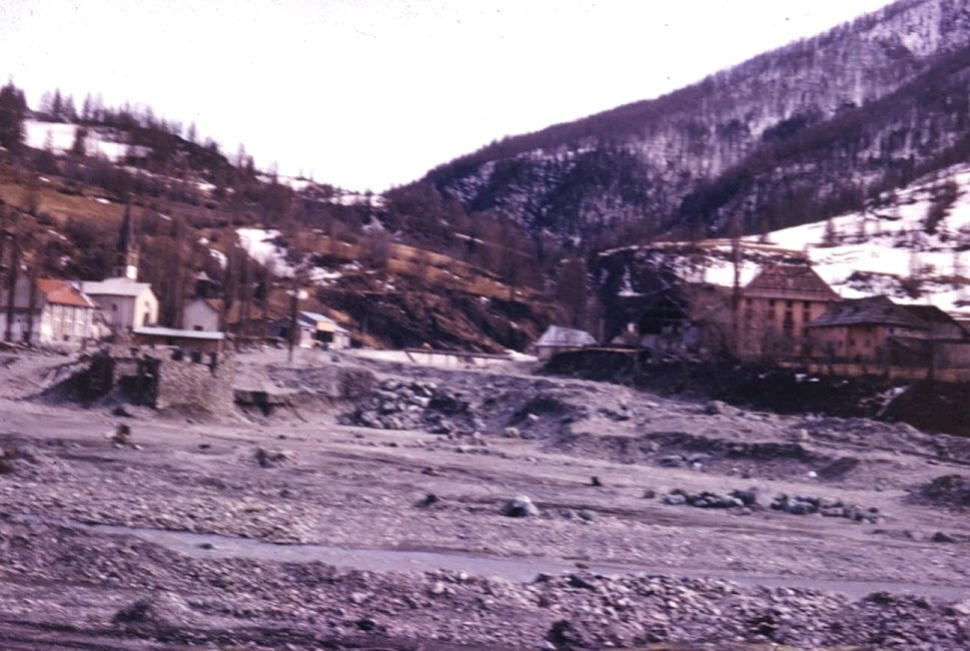
View upstream in 1962 of the alluvial fan in which a village stood before the catastrophic 1957 Guil Valley flood (photo by Ian Douglas)

Perhaps my most vivid memory of France in 1962 was seeing this village in the valley of the River Guil in the French Alps where a torrential mudflow had destroyed nearly all the village on this alluvial fan a few years earlier (Tricart 1960, p. 278). In 1957, a huge debris flow descended this little valley and swept away most buildings that stood in the foreground of this image (Fig. 10). It demonstrates the importance of siting human settlements in suitable places, and the value of applied geography, in this case geomorphological mapping, in deciding where those suitable places are.

### 1969 Urban erosion in Kuala Lumpur, Malaysia

**Fig. 11:**
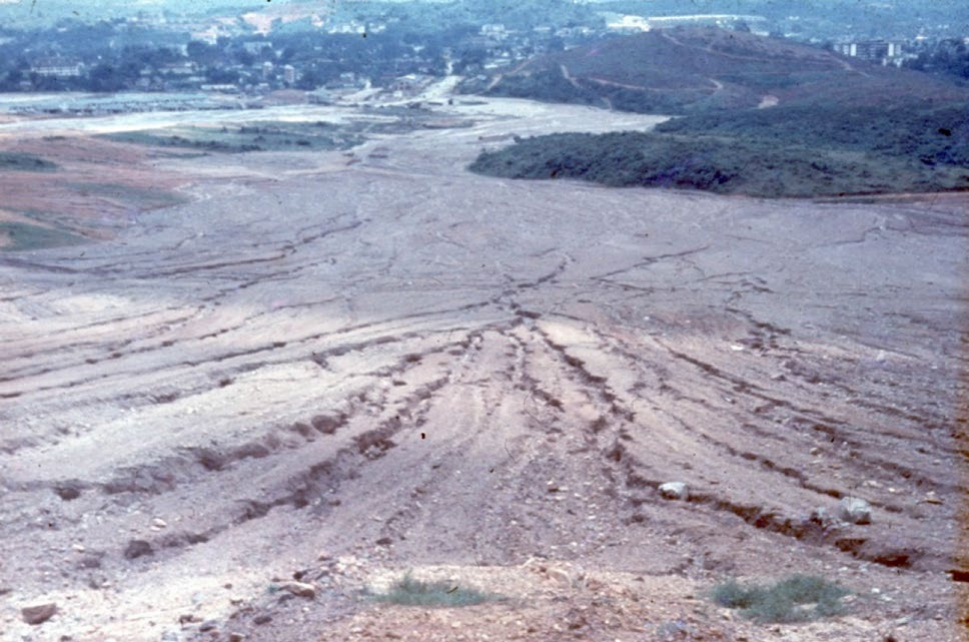
View from the Damansara Heights construction site towards the centre of Kuala Lumpur (top left) in 1969 showing rapidly developing erosion gullies that led to blocked channels and flooding downstream every time heavy rain occurred (photo by Ian Douglas)

This is land at Damansara Heights in Kuala Lumpur that had been cleared of vegetation ready for development in the late 1960s, but left bare for many months, so that every tropical thunderstorm downpour washed away the deep soil and weathered rock (Fig. 11). The sediment was carried down to a small stream at the lowest pot of the site. That stream was partially blocked by the sediment and had so much storm runoff that it overflowed, flooding nearby houses and the main road linking the centre of Kuala Lumpur to its satellite town, Petaling Jaya. This really drove home to me how important applied physical geography is in the urban environment.

### 1996 Extreme flood on the Sungai Segama, Sabah, Malaysia

**Fig. 12:**
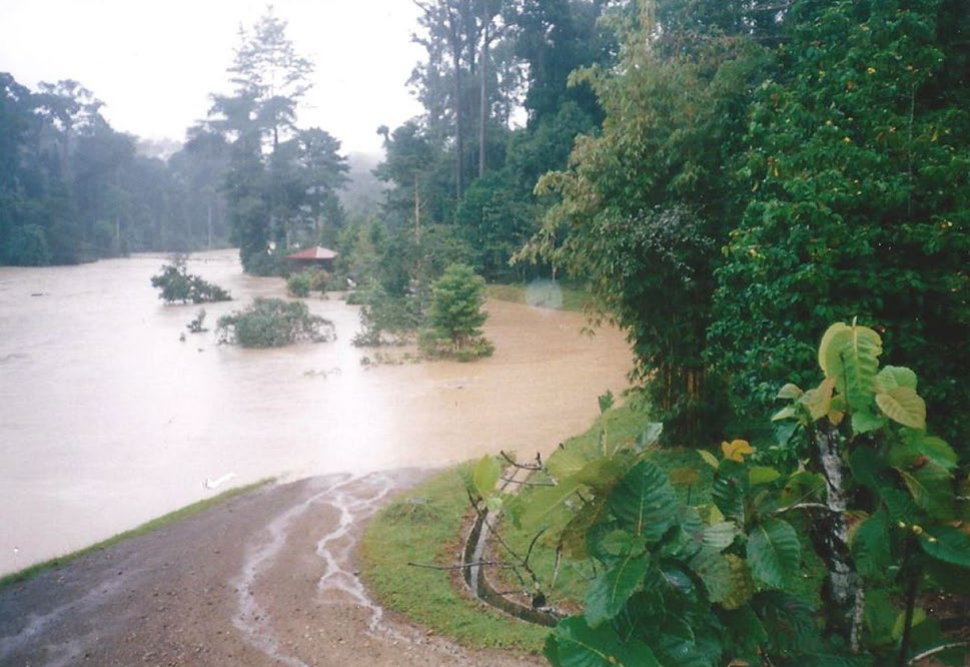
Flood on the Sungai (River) Segama at the Danum Valley Field centre 19th January 1966. The access road to the Centre is in the foreground. A flood of this magnitude might have a recurrence interval of 1 in 10 years (photo by Ian Douglas)

On January 19th 1996 at Danum Valley Field Centre, Sabah, Malaysia after 177 mm of rain in 11 h, the Sungai Segama flooded the lower part of the field centre, cutting its access road (Fig. 12).

This big moment came when I awoke in the night to the pounding of extremely heavy rain on the roof at Danum. As soon as there was enough light, I took a sediment sampler down to the suspension footbridge across the Segama River which clearly rising rapidly and already carrying much woody debris. I began taking suspended sediment samples every hour until nightfall. I continued the next morning. Calculation of the total load carried that day showed that in single 24-hour period the river had carried more sediment than in the previous 4 years. This showed the power of an extreme event in the tropical rain forest.

We walked up to the head waters of our reference small catchment and followed its headwaters right to the divide. Tiny rivulets had developed everywhere in the litter on the forest floor. Water was running off the leaves of trees like water off the roof of a house. There was no infiltration because the ground was already saturated. Every drop reaching the ground would be conveyed along the temporary channels to the stream and out to the Segama River. There is a big lesson here for those who advocate tree planting to reduce flooding. Yes, in moderate storms, trees will retain and impede water flows and lessen minor floods. But, even in dense jungle, in extreme rainfalls, such as this 1 in 10-year fall at Danum, the whole system can become so saturated that water runs off the vegetation just as quickly as it runs of the roofs of buildings. Forests help to reduce river flows from moderate storms but not from the really big, rare, super storms. A second lesson is that although they flow with water nearly all the time, small rainforest streams can vary greatly in length of channel flow. Our headwater stream dried up completely for the uppermost 1.5 km of its defined channel in drought, but in this January 1996 storm it flowing length was all that 1.5 km plus another 0.7 km of channel through the forest floor litter. In a way, the behaviour of these streams is somewhat like a desert arroyo or wadi that only flows during rare storms. In both cases most of the work of moving sediment happens in the rare big events. Far from being a steady, regulating, water-conserving environment, the tropical forest is an event-driven hydrological system, subject to long periods of relative inactivity and sudden dramatic changes during rare large storms.

## A grateful epilogue

**Fig. 13:**
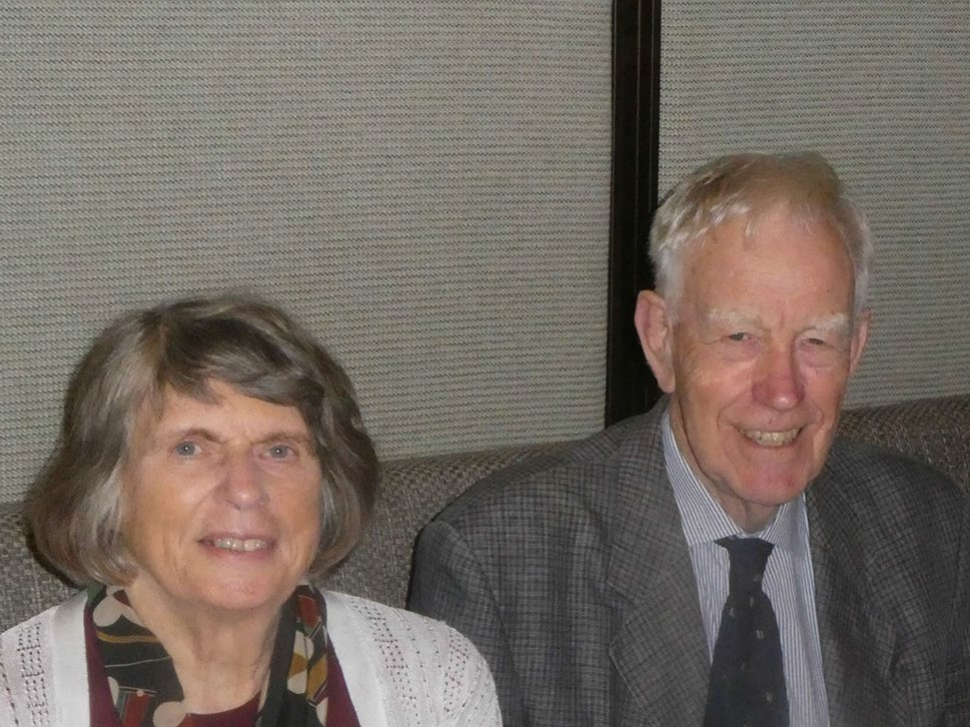
Maureen and Ian Douglas in Kuala Lumpur in 2019

I have had a wonderful 55 years doing all the things mentioned above. I realise I have been fortunate in the opportunities I have had and the wonderful people I have been able to work with. I owe and enormous debt to Maureen, my wife of 56 years who started married life doing field work in North Queensland with me, sitting on river banks recording my stream flow observations and who more than 55 years later will critically read what I have written here and tell me what needs changing or correcting (Fig. 13). She has shared so much with me, yet became a social scientist in her own right, doing for more for other people than I possibly could.

I know I have unfinished tasks awaiting me. They include preparing articles on mangroves for the CHEC Journal “Human Ecology”; finishing a guide on Chorlton for the Manchester Geographical Society; finishing a book on the hydrological work at Danum Valley; revising an article on medieval cities and the environment; and getting the Handbook of Urban Ecology through the publisher’s production process. I am thus at times juggling my interests; sustaining both the scientific trend in the rainforest work and the practitioner trend in the urban environment work. I hope that what I do is constructive and helpful.

Our rapidly changing world is full of exciting challenges. The COVID-19 pandemic indicates how an extreme event may pose a global problem, but local preparedness varies and the burdens fall unequally across societies. We have to rise to these challenges and see the relevance of our work to both the global and the local situation. Not many of us make world-shattering discoveries or policies, but we can make a difference by what we choose to work on and how we can use that work to create opportunities for others. The collective success of the many scientists at Danum Valley, Sabah, Malaysia has contributed to higher protection of rainforests in the Yayasan Sabah Concession area. The University of New England geographers’ mapping of wilderness in New South Wales helped in temperate rainforest areas be designated National Parks. We can make a difference through active geography and socio-ecological practice.

## Appendix 1: Key steps during my preparations for, and along, my 55-year journey

**Table:**

Years	Institution	Role	Key activity	Key persons
1940–48	Preston Park Primary School, London	Pupil		Miss Gifford (headmistress)
1948–55	Merchant Taylors School, London	Pupil		Joe Gerber and Charles Hull (Geography Teachers)
1956–58	Compulsory National Service in Royal Artillery	Bombardier	Technical Assistant	Tony Schärer, fellow Bombardier, who also read geography at Oxford in 1958
1958–63	Balliol College, Oxford University	Student in the Honours School of Geography	B.A. and B.Litt. degrees	Martyn Webb (college tutor); Marjorie Sweeting (graduate supervisor); Edmund W. Gilbert (Head of School)
1963–66	Australian National University	Ph.D. student in the Research School of Pacific Studies	Fieldwork in north Queensland and Southeastern New South Wales	Joe Jennings (Ph.D. Supervisor); Donald Walker; Harold Brookfield; Oskar Spate (Head of Department); Harold Scholz (Queensland Irrigation and Water Supply Commission).
1966	Universiti Malaya	Visiting Lecturer	Research into rainforest and urban erosion	Hamzah Sendut (Head of Geography); Jim Jackson (later a colleague at Hull)
1966–71	University of Hull	Lecturer	Teaching and research	Harry Wilkinson (Head of Department)
1969–70	Universiti Malaya	Visiting Lecturer	Research into rainforest and urban erosion	Roy Morgan (Lecturer and Ph.D. candidate: later Professor at Cranfield University)
1971–78	University of New England, Australia	Professor of Geography	Research, Graduate Supervision, Teaching, Head of Department for 4 years	Gilbert Butland (Foundation Professor) David Lea, Ian Jackson and Jim Walmsley (colleagues)
1979–97	University of Manchester	Professor of Physical Geography	Research, Graduate Supervision, Teaching, Head of Department for 6 years	Brian Rodgers (fellow Professor); Martin Harris (Vice-Chancellor); Jack Zussman (Professor of Geology)
1991	Université Paris 1 Panthéon - Sorbonne	Professeur Invité	Teaching	Alain Godard (Professor of Geomorphology)
1997 to present	University of Manchester	Emeritus Professor	Teaching and Graduate Supervision until 2008. Research continues	John Handley (Professor of Landscape); Joe Ravetz (Senior Research Fellow); Nigel Lawson (Research Fellow) colleagues on urban ecology
1997	Mekong River Commission, Bangkok	Consultant	Assessment of erosion and sedimentation projects	Tran Van Phuc (Associate Environmentalist) and Keobang Keola (Project Officer)
1997–8	Université Paris 1 Panthéon - Sorbonne	Professeur Invité	Teaching	Lucien Faugères (Professor of Physical Geography)
2000–6	Natural Environment Research Council	Science Co-ordinator LOCAR Programme	Delivering integrated research outcomes	Harold Wheater (then Professor of Hydrology at Imperial College);

## Appendix 2: Ongoing and previous commitments to organisations and work outside the University

**Table:**

Organisation	Date joined	Highest position	Key activities
British Society for Geomorphology	1968	Chairman 1980–1 Fellow since 2013	1981 Organised Annual Meeting in Manchester: symposium on tropical geomorphology 1985 Organised First International Conference on Geomorphology
British Hydrological Society	1983	Member	Organising Tropical Hydrology Session for 2021 annual symposium
Manchester Geographical Society	1982	President 1984	2020 Currently preparing contributions to Exploring Greater Manchester
UK Urban Forum	1992	Chairman 1993–98 and 2010–12	2020 Awaiting publication of the Second Edition of the Routledge Handbook on Urban Ecology: a project of the Forum
Commonwealth Human Ecology Council (CHEC)	1993	Chairman Governing Board 1999–2009	Projects included: EU funded Asia Pro-Eco Project in Ahmedabad, India; Gender mainstreaming in Water Resources Management, Uganda; Commonwealth-wide mangrove conservation
Society for Human Ecology (SHE)	2005	President 2009–11	2005 Keynote address to Annual Conference 2009 Organised International Conference with CHEC and German Society for Human Ecology in Manchester
Royal Geographical Society (with IBG) (RGS)	1953	Council Member 1991–4	Contributed to *Geographical Journal* and the *Expedition Planners handbook & directory*
Institute of Australian Geographers (IAG)	1966	President 1978	Townsville IAG meeting
Australian and New Zealand Association for the Advancement of Science (ANZAAS)	1964	President Section 21 (Geographical Sciences) 1973	Keynote address on the future of Australian rainforests
British Association for the Advancement of Science (BAS)	1967	President Section E Geography 1993	Organised sessions on tropical rainforest and on the urban environment
Scientific Committee on Problems of the Environment (SCOPE) of the International Council for Scientific Unions (ICSU)	1994	Treasurer 2002–9 Chair UK SCOPE Working Party (Royal Society)	Led projects on Earth Surface Processes, Mining and Urban Development (ESPROMUD) and Peri-urban Environmental Change (PU-ECH)
International Council for Ecopolis Development (INTECOPOLIS)	2007	President 2007–14	Chaired conferences: liaised with international partners and Chinese host city personnel.

## Footnotes

1 I use questions Wei-Ning Xiang asked as the section titles throughout the essay, and develop titles myself for subsections. In Appendices, I tabulate chronologically key steps during my preparations for, and along, my 55-year journey, and the ongoing and previous commitments to organisations and work outside the University. For definitions of socio-ecological practice and socio-ecological practice research, see Xiang (2019).

2 “L’idéalisme est un luxe que nous pourrons de moins en moins nous permettre. Le temps presse. Les sols s’érodent, tandis que les hommes ont faim. Des taches immenses attendant les générations prochaines de géomorphologues.” (Cailleux and Tricart 1961, p.).

3 “D’abord active, vivante, applicable et appliquée” (Schmitz 2003, p.).
